# Hypodontia phenotype in patients with epithelial ovarian cancer

**DOI:** 10.2478/raon-2014-0034

**Published:** 2015-03-03

**Authors:** Anita Fekonja, Andrej Cretnik, Danijel Zerdoner, Iztok Takac

**Affiliations:** 1 Department of Orthodontics, Health Centre Maribor, Maribor, Slovenia; 2 Medical Faculty, University of Maribor, Maribor, Slovenia; 3 Department of Maxillofacial and Oral Surgery, Teaching Hospital Celje, Celje, Slovenia; 4 Department of Gynaecologic and Breast Oncology, Clinical Department of Gynaecology and Perinatology, University Clinical Centre Maribor, Maribor, Slovenia

**Keywords:** hypodontia, epithelial ovarian cancer, risk factor, early stage diagnosis

## Abstract

**Background:**

Ovarian cancer is usually diagnosed in an advanced stage and the present clinical and diagnostic molecular markers for early OC screening are insufficient. The aim of this study was to identify potential relationship between the hypodontia and epithelial ovarian cancer (EOC).

**Patients and methods:**

A retrospective study was conducted on 120 patients with EOC treated at the Department of Gynaecologic and Breast Oncology at the University Clinical Centre and 120 gynaecological healthy women (control group) of the same mean age. Women in both groups were reviewed for the presence of hypodontia and the patients with EOC also for clinicopathological characteristics of EOC according to hypodontia phenotype.

**Results:**

Hypodontia was diagnosed in 23 (19.2%) of patients with EOC and 8 (6.7%) controls (p = 0.004; odds ratio [OR] = 3.32; confidence interval [CI], 1.42–7.76). There was no statistically significant difference in patients with EOC with or without hypodontia regarding histological subtype (p = 0.220); they differed in regard to FIGO stage (p = 0.014; OR =3.26; CI, 1.23–8.64) and tumour differentiation grade (p = 0.042; OR = 3.1; CI, 1.01–9.53). Also, bilateral occurrence of EOC was more common than unilateral occurrence in women with hypodontia (p = 0.021; OR = 2.9; CI, 1.15–7.36). We also found statistically significant difference between the ovarian cancer group and control group in presence of other malignant tumours in subjects (p < 0.001).

**Conclusions:**

The results of the study suggest a statistical association between EOC and hypodontia phenotype. Hypodontia might serve as a risk factor for EOC detection.

## Introduction

Ovarian cancer is the most fatal malignancy of the female genital tract in the Western world.^1^–^3^ In Slovenia, ovarian cancer is the eighth overall and the second most common gynaecologic cancer (after endometrial one).^1^ Nonspecific symptoms, lack of reliable (bio)markers, frequent diagnosis in advanced stage (approximately 75–80% of ovarian cancers are diagnosed in stage III–IV), and the presence of drug-resistant histologic types limit the long-term cure rates and prognosis of the disease.^4^–^7^ Ovarian cancer is usually seen in peri- or postmenopausal women.^1^,^5^ Multiple reproductive, demographic and lifestyle factors are known to influence a woman’s risk of developing this cancer, but, the strongest known risk factor is a positive family history.^5^,^8^–^11^ Approximately 5–10% of ovarian cancer occurs in those known to carry BRCA mutations, depending on the population or ethnic group. In women with a family history of breast or ovarian cancer and a known BRCA mutation, the cumulative lifetime risk of developing the disease has been estimated to be approximately 40–50% for BRCA1 and 20–30% for BRCA2, compared with an approximate 1.6% lifetime risk in the general population.^11^,^12^ In hereditary nonpolyposis colorectal cancer families (also known as Lynch II syndrome) lifetime risk of ovarian cancer in carriers of mismatch repair gene mutation is around 6.7–12%.^5^,^11^ However, these known gene faults do not account for all of the inherited risk in women with a family history of ovarian cancer. Other »high risk« ovarian cancer genes may exist, although mutations in these genes are probably less common than BRCA1 and BRCA2. It is likely that much of the remaining familial risk is due to a combination of several genes that each individual contributes, rise to a low or moderate risk.^11^

In our (dental) clinical practice we have seen more frequent positive self-reported family history of some disease (colorectal cancer, ovarian cancer, thyroid disease) in patients with hypodontia than those without hypodontia.

A congenital anomaly affecting the formation of the dentition that results in a reduction of the usual number of the human deciduous dentition (20 total teeth in both jaws) and/or the permanent dentition (a total of 32 teeth in both jaws) is commonly referred to as aplasia. The term hypodontia is used when one to five teeth, excluding the third molar, are absent. The condition, when more than five teeth, excluding the third molar, are absent, is called oligodontia. Anodontia is an extreme case, denoting the complete absence of teeth.^13^

Dental agenesis is an important clinical and public health problem. Patients with hypodontia may suffer from a reduced chewing ability, inarticulate speech, and an unfavourable aesthetic appearance. Comprehensive management often requires a multidisciplinary approach. The reported prevalence of hypodontia in the permanent dentition in population vary from 2.6% to 11.3%^14^,^15^, being lower in North America than in European countries.^16^ In the Caucasian population the most frequently affected teeth were lower second premolars, followed by the maxillary lateral incisors and the upper second premolars. Most studies showed a higher prevalence of hypodontia in females.^15^–^17^

The aetiology of hypodontia is multifactorial with major genetic and environmental factors. Hereditary or family history has been suggested as the primary cause for hypodontia.^16^ In familial hypodontia, the type of inheritance in the majority of families seems to be autosomal dominant with incomplete penetrance and variable expression. Sex-linked and polygenic models of inheritance are also possible.^18^ Various genes including PAX9, MSX1 and AXIN2 have been implicated in the aetiology of hypodontia.^19^,^20^ The AXIN2 gene regulates the *Wnt* signalling pathway, which plays an important role in the cellular proliferation, differentiation and morphogenesis of most organs, and control of ß-catenin stability is central to *Wnt* signalling. Defects that interfere with ß-catenin regulation have been reported in various human cancers. The *Wnt* pathway has been well studied in a number of cancers where ß-catenin mutations could be identified (like in colorectal cancer).^20^–^22^ In ovarian cancer, however, the detection of a high-rate of ß-catenin mutations (approximately 40%) was confined to the endometrioid subtype of epithelial ovarian cancer.^23^

On the other hand, it has been suggested that a major hereditary contribution to cancer is given by largely unknown alleles of low penetrance and some of these alleles may also contribute to the background of tooth agenesis. It is conceivable that defects in other genes, in addition to AXIN2, involved in *Wnt* signalling and regulation of ß-catenin level may also show a link between tooth agenesis and cancer predisposition. However, relationship among hypodontia and other pathological conditions, such as the colorectal cancer, has already been shown^21^ and if a link between hypodontia and ovarian cancer could be established, this could serve as a potential risk factor for detection of ovarian cancer, particularly in earlier stages.

The aim of the study was to identify a possible correlation between hypodontia phenotype and epithelial ovarian cancer (EOC). Therefore, this study sought to evaluate and compare the International Federation of Gynecology and Obstetrics (FIGO) stage, the histologic subtype, the grade, the side of tumour according to the presence of hypodontia phenotype. We also analysed the family history of ovarian cancer and other cancer in both groups.

## Patients and methods

The study included 120 consecutive patients with histologically proven EOC who were treated at the Department of Gynaecologic and Breast Oncology at the University Clinical Centre Maribor, and 120 gynaecological healthy women who had given their formal consent after receiving an explanation about the aim of this study. The study was approved by the Ethics Committee at the Ministry of Health of Slovenia.

Clinic pathological characteristics of patients, including age at diagnosis of the EOC, definitive FIGO stage, postoperative histologic subtypes, postoperative tumour grade, and side of tumour (unilateral/bilateral) were collected from medical records. Tumours were classified as FIGO stage, using the grading system proposed by Benedet.^24^

One-hundred-twenty gynaecological healthy women (of the same mean age as study group) represented control group, which was confirmed by general gynaecological examination, including the transvaginal ultrasound. In the control group exclusionary criteria included women with any ovarian abnormalities as assessed by transvaginal ultrasound to detect abnormal ovarian morphology, which is an indicator of a potential malignancy. Dental examination for all subjects was conducted at the Department of Orthodontics in Health Centre Maribor. A tooth was registered as congenitally missing when no trace could be found on the radiograph and the dental treatment records confirmed that the tooth had not been extracted. Third molars were not included in the research. Other teeth abnormalities, such as microdontia, were excluded from criteria to denote hypodontia pheno-type. Women with dentures or women in whom we can not determine the cause of missing teeth were not included in the study. No subjects had cleft lip/palat or any syndrome. We interviewed each subject about her family history of cancer. If at least one family member had had cancer, that participant was considered to have a family history of cancer. Data were collected between December 1^st^, 2010 and October 24^th^, 2013 and analysed statistically.

### Statistical analysis

Descriptive statistics were used to present baseline characteristics. Unpaired (two-sample) t-test at a significance level of < 0.05 was used to assess the differences in age between the EOC patients and the control group. We analysed the difference in prevalence rates of hypodontia among the EOC patients and the gynaecological healthy women (control group), and the difference in FIGO stage, histologic subtypes, tumour differentiation grades, and the tumour bilateralism between EOC patients with and without hypodontia using chi-square test or Fisher exact test with a p value of < 0.05 as a standard for a statistically significant difference. To determine the relationship between hypodontia and ovarian cancer we used logistic regression model. The results were presented as odds ratios (OR) with corresponding 95% confidence interval (CI).

## Results

The mean (standard deviation [SD]) age of the EOC patients at time of diagnosis was 53.1 (11.1) with a median of 53 years, and mean (SD) age of the control group was 53.3 (10.7) with a median of 54 years (p = 0.878).

In 120 patients with EOC, hypodontia pheno-type was found in 23 (19.2%) patients and in eight out of 120 healthy individuals (6.7%). The difference between these two hypodontia rates was statistically significant (p = 0.004). The data also showed that the OR was 3.32 (95% CI, 1.42–7.76).

In Table 1 further analysis of the women with EOC (study group) with or without hypodontia is presented (FIGO stage and histology). Statistically significant difference was found in FIGO stage (p = 0.014; OR = 3.26; 95% CI, 1.23–8.64). Histopathological analysis showed predominantly serous subtype of epithelial ovarian cancer in women with and without hypodontia and no significant differences between groups regarding definitive histology (p = 0.220).

Most (64.7%) of the invasive tumours in patients with hypodontia were poorly differentiated tumours, 17.6 % moderately and 17.6 % highly differentiated, whether in women without hypodontia poorly differentiated, moderately differentiated, and highly differentiated ones were observed almost at the same range (37.1 %, 37.1 %, and 25.8 %). In patients with epithelial ovarian cancer and hypodontia, we observed a significantly higher proportion of poorly differentiated tumours (high-grade tumour malignancy, gradus 3) than in patients without hypodontia (64.7 % in 37.1 %, p = 0.042). Patients with hypodontia have 3.1 (95% CI, 1.01–9.53) times the odds ratio as patients without hypodontia of having poorly differentiated tumours (gradus 3).

In 56.5% of our patients with hypodontia and in 30.9% of patients without hypodontia ovarian cancer was bilateral (p = 0.021; OR = 2.9; 95 % CI, 1.15–7.36) (Table 2).

We also found statistically significant difference in presence of other malignant tumours in subjects between the ovarian cancer group and control group (p < 0.001). Most commonly reported additional malignant tumours in study group were breast, uterus, intestine and colon, and thyroid carcinoma and breast carcinoma in control group, but without statistically significant difference in tumours types between groups. Otherwise no other statistically significant differences were found in family history (Table 3).

## Discussion

Ovarian cancer, known as the silent killer in women, is difficult to diagnosis due to a lack of effective early screening markers for this disease. Without improvements in the current early detection protocols, over 75% of women diagnosed with ovarian cancer will be identified in late stage of disease with a significantly reduced chance of survival.

Lopes *et al*.^25^ reported that anomalies of the teeth may be present in many diseases and dentists may be the first to notice them particularly through the preventive children screening programmes (obligatory in Slovenia). Previous studies have demonstrated that the genes that control the tooth development may have an important function in other organs and cancer diseases.^21^,^26^ Zhai *et al*.^27^ indicated in their study that ß-catenin and TCF plays a vital role in the activation of AXIN2 expression in colon and ovarian cancer cells. Lammi *et al*.^21^ reported about evidence of the expression of association AXIN2 in colorectal tissue leading to carcinoma and hypodontia in a Finnish family. It is interesting that one gene mutation can cause tooth agenesis and predispose to colorectal cancer. Although 10% of ovarian cancer is familial and the majority of the inherited types are related to mutations in the BRCA1 and BRCA2 genes^8^, other unknown genetic mutations may play a crucial role as well.

We found only one paper in the literature showing possible association between teeth anomalies and epithelial ovarian cancer (EOC) by Chalothorn *et al*.^28^ Authors found low prevalence of hypodontia in control group (3%; 3/100), despite they included patients with microdontia and hypodontia and 20% prevalence (10/50) in patients with EOC with statistically significant difference between groups. In our study only patients with hypodontia were included. The present study revealed hypodontia phenotype prevalence in 19.2% and 6.7% for the EOC group and control group, respectively.

**FIGURE 1.**
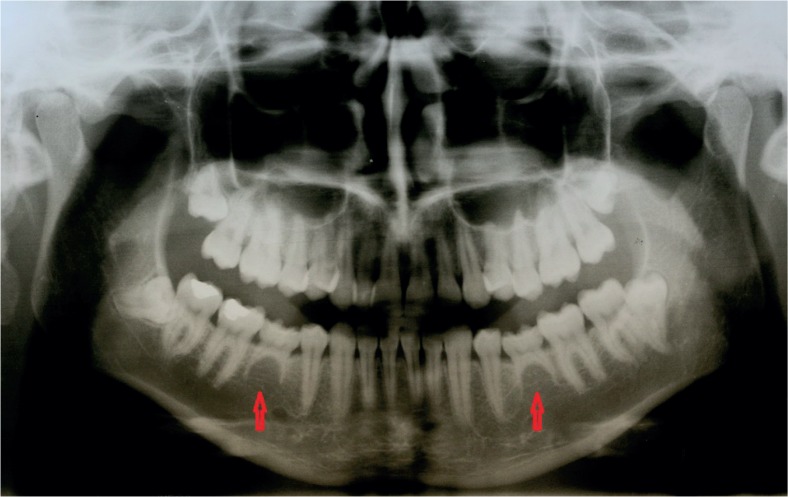
Hypodontia of both lower second premolars (persistent deciduous second molars).

The prevalence of hypodontia in population varies quite a lot. Mattheeuws *et al*.^16^ reported prevalence of hypodontia in meta-analysis in the Caucasian population within 3.9–10.1%. Study by Fekonja^29^ showed 6.9% (7.8% in female and 5.9% in male) hypodontia prevalence in Maribor population (Slovenia). Results of our study show higher hypodontia prevalence in women with EOC, while the prevalence of control group was similar to Slovenian population.^29^ As hypodontia can be diagnosed early in the life (by radiographs as early as age nine), a possible association between hypodontia pheno-type and EOC could serve as the earliest possible quantifying risk factor for the EOC, before the development of clinical evident disease (Figure 1).

In the study we also evaluated the FIGO stages, the histological subtypes, and tumour grades. Other authors reported that over 75% of patients are diagnosed at the advanced stage, when cancer has already metastasized, because of non-specific clinical symptoms.^4^–^7^ In our study 69.6% patients with hypodontia and 41.2% patients without hypodontia were diagnosed in stage II, III, or IV. Screening protocols for women with hypodontia could be of a great help in detecting ovarian cancer in earlier stage with potential better curing possibilities and prognosis.

As reported by other authors^30^, serous carcinoma is the most common histological subtype of ovarian cancer, what was also found in our study (78.3% ovarian serous carcinoma in patients with hypodontia corresponding to 64.9% in the group without hypodontia).

But^31^ reported that 46 % EOC were poorly differentiated, 38.1% moderately and 15.9% highly differentiated tumours. In our study also predominated poorly differentiated tumours (43%), followed by moderately (33%) and highly (24%) differentiated. According to the hypodontia we founded the majority (64.7%) of poorly differentiated tumours in patients with hypodontia*,* whether in women without hypodontia moderately differentiated, highly differentiated and poorly differentiated ones were observed almost at the same range.

In our study we also evaluated the uni-or bilateralism of EOC occurrence. In 23 patients with hypodontia the tumour was bilateral in 13 patients (56.5%) and unilateral in 10 patients (43.5%). In 97 patients without hypodontia the tumours were bilateral in 30 subjects (30.9%) and unilateral in 67 subjects (69.1%). We can conclude that there might be a trend toward more frequent bilateralism in patients with hypodontia.

In our study there was statistically significant difference in patients with EOC with or without hypodontia regarding FIGO stage (p = 0.014; OR = 3.26; CI, 1.23–8.64), tumour differentiation grade (p = 0.042; OR = 3.1; CI, 1.01–9.53), and bilateralism (p = 0.021; OR = 2.9; CI, 1.15–7.36), but no statistically significant difference was found in histological subtype (p = 0.220). However, in the multivariate model the relationship of hypodontia with the FIGO stage, tumour differentiation grade, bilateralism and histological subtype of EOC could not be confirmed (p = 0.368). Chalothorn *et al*.^28^ did not report of any clinicopathological characteristics of the tumour in their study so we can not compare their findings with our results.

Kücher *et al*.^26^ reported that individuals with hypodontia had an increased risk of having a family history of breast, ovarian, and cervical uterine cancer. In our study we confirmed the correlation between hypodontia and other malignant tumours, but no significant difference between hypodontia and family history of ovarian or other malignant tumours.

In conclusion, this study indicated statistically significant association between EOC and hypodontia phenotype. Congenital absence of permanent teeth has direct visual implication and could identify patients with hypodontia phenotype and serve as a possible risk factor for EOC detection. The results of this study suggest that more research in this topic is needed to be able to identify genetic markers, which, combined with the presence of missing teeth, could identify women of greatest risk for developing EOC in their lifetime at a much younger age than previously possible.

## Figures and Tables

**TABLE 1.** FIGO stage and histologic types of tumours in study groups

**CHARACTERISTICS**	**WITH HYPODONTIA**	**WITHOUT HYPODONTIA**
FIGO STAGE^*^		
Stage I	7 (30.4%)	57 (58.8%)
Stage II	4 (17.4%)	7 (7.2%)
Stage III	8 (34.8%)	28 (28.9%)
Stage IV	4 (17.4%)	5 (5.1%)
HISTOLOGIC SUBTYPES^**^		
Papillary serous adenocarcinoma	18 (78.3%)	63 (64.9%)
Mucinous cystadenocarcinoma	1 (4.3%)	15 (15.5%)
Endometrioid carcinoma	3 (13.1%)	13 (13.4%)
Clear cell carcinoma	1 (4.3%)	0 (0%)
Malignant Brenner tumour	0 (0%)	3 (3.1%)
Mixed tumour	0 (0%)	2 (2.1%)
Undifferentiated carcinoma	0 (0%)	1 (1.0%)

* *χ*^2^ = 8.826; *p* = 0.014

** *χ*^2^ = 7.853; *p* = 0.220

**TABLE 2.** Distribution of tumors according to its presence unilaterally or bilaterally

**Study group**	**Unilaterally**		**Bilaterally**
	**Right**	**Left**	
With hypodontia (n=23)	7 (30.4%)	3 (13.1%)	13 (56.5%)
Without hypodontia (n=97)	34 (35.1%)	33 (34%)	30 (30.9%)

χ^2^ = 5.296; *p*= 0.021

**TABLE 3.** Presence of malignant tumours in subjects and family members according to hypodontia

	**Study group**	**Control group**	**P-value**
All other malignant tumours in subjects	32 (26.7%)	9 (7.5%)	< 0.001
Family history of ovarian cancer	9 (7.5%)	7 (5.8%)	0.617
Family history of other malignant tumour	65 (54.2%)	62 (51.7%)	0.698
